# Apolipoprotein L genes are novel mediators of inflammation in beta cells

**DOI:** 10.1007/s00125-023-06033-z

**Published:** 2023-11-04

**Authors:** Miriam Paz-Barba, Amadeo Muñoz Garcia, Twan J. J. de Winter, Natascha de Graaf, Maarten van Agen, Elisa van der Sar, Ferdy Lambregtse, Lizanne Daleman, Arno van der Slik, Arnaud Zaldumbide, Eelco J. P. de Koning, Françoise Carlotti

**Affiliations:** 1 Department of Internal Medicine, Leiden University Medical Center, Leiden, the Netherlands; 2 Department of Cell and Chemical Biology, Leiden University Medical Center, Leiden, the Netherlands

**Keywords:** Apolipoprotein L, Beta cells, Human islets, Inflammation

## Abstract

**Aims/hypothesis:**

Inflammation induces beta cell dysfunction and demise but underlying molecular mechanisms remain unclear. The apolipoprotein L (APOL) family of genes has been associated with innate immunity and apoptosis in non-pancreatic cell types, but also with metabolic syndrome and type 2 diabetes mellitus. Here, we hypothesised that *APOL* genes play a role in inflammation-induced beta cell damage.

**Methods:**

We used single-cell transcriptomics datasets of primary human pancreatic islet cells to study the expression of *APOL* genes upon specific stress conditions. Validation of the findings was carried out in EndoC-βH1 cells and primary human islets. Finally, we performed loss- and gain-of-function experiments to investigate the role of *APOL* genes in beta cells.

**Results:**

*APOL* genes are expressed in primary human beta cells and *APOL1*, *2* and *6* are strongly upregulated upon inflammation via the Janus kinase (JAK)−signal transducer and activator of transcription (STAT) pathway. *APOL1* overexpression increases endoplasmic reticulum stress while *APOL1* knockdown prevents cytokine-induced beta cell death and interferon-associated response. Furthermore, we found that *APOL* genes are upregulated in beta cells from donors with type 2 diabetes compared with donors without diabetes mellitus.

**Conclusions/interpretation:**

APOLs are novel regulators of islet inflammation and may contribute to beta cell damage during the development of diabetes.

**Data availability:**

scRNAseq data generated by our laboratory and used in this study are available in the Gene Expression Omnibus (GEO; www.ncbi.nlm.nih.gov/geo/), accession number GSE218316.

**Graphical Abstract:**

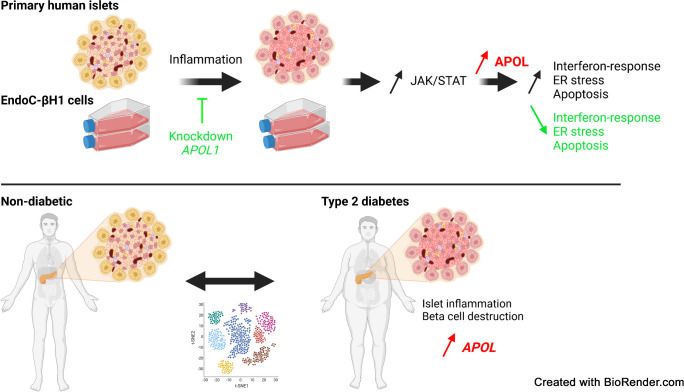

**Supplementary Information:**

The online version of this article (10.1007/s00125-023-06033-z) contains peer-reviewed but unedited supplementary material.


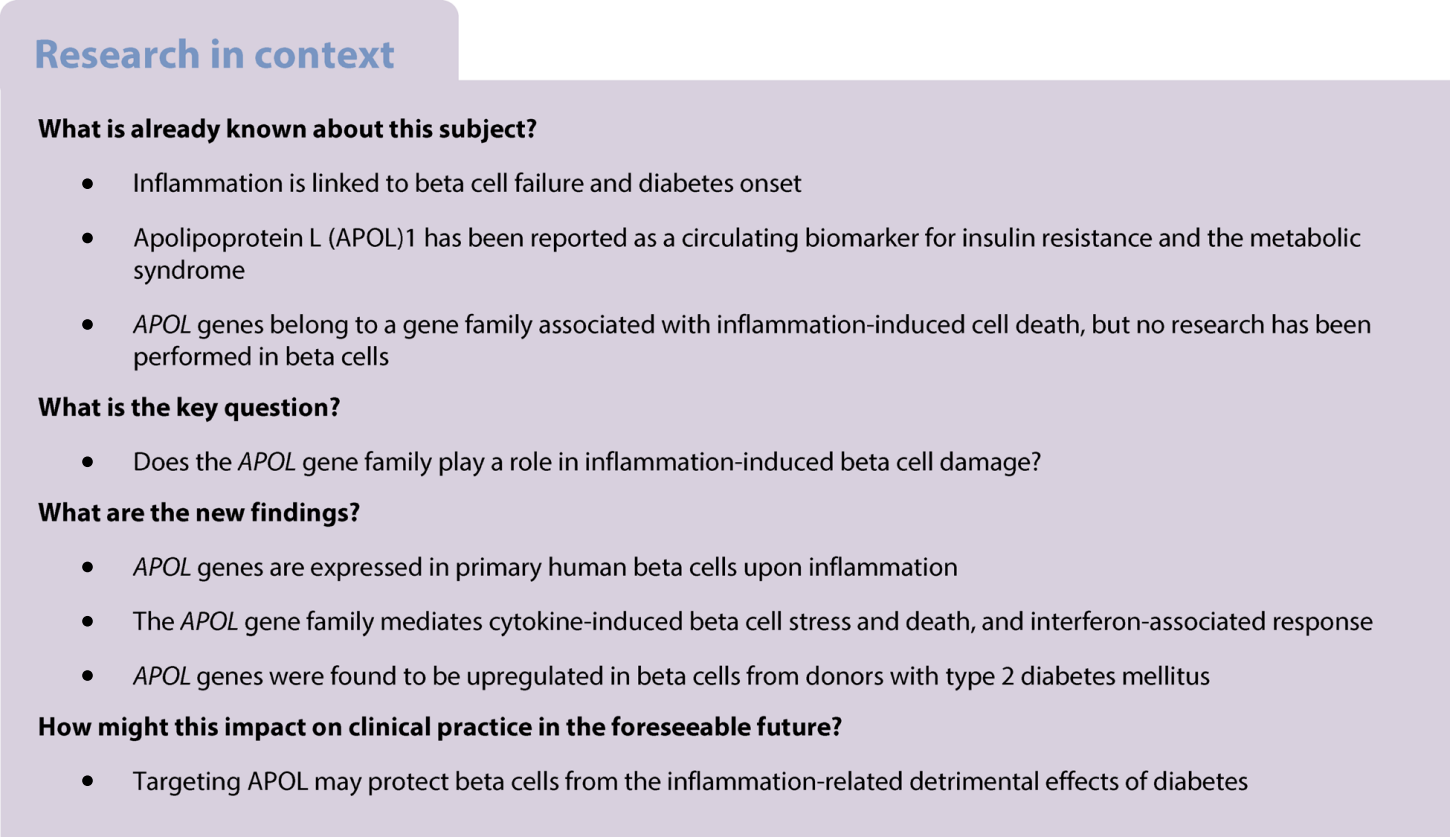


## Introduction

Inflammation is one of the most studied causes of beta cell failure. Specifically, exposure to proinflammatory cytokines has been shown to impair beta cell function and increase beta cell death [1–3]. Cytokine-induced beta cell damage is regulated via a broad spectrum of pathways. IL-1β can induce inflammation and eventually cell death via NF-κB activation and expression of proinflammatory genes (e.g. *iNOS* [also known as *NOS2*] and *CXCL10*) [4]. IFNγ activates the Janus kinase (JAK)−signal transducer and activator of transcription (STAT) pathway, which leads to STAT translocation to the nucleus and upregulation of target inflammation genes (e.g. interferon regulatory factors [IRFs]) [5, 6]. In addition, the combination of IFNγ + IL-1β can also trigger cell death via the endoplasmic reticulum (ER) stress pathway and the increase in the pro-apoptotic genes *ATF3* and *CHOP* [7, 8]. In a vicious cycle, the cytokine-induced expression of proinflammatory genes via the different pathways mentioned can also lead to the amplification of inflammatory signalling and contribute to cell death.

Targeting inflammation-related pathways has already shown promise for protecting beta cells in the context of both type 1 diabetes mellitus [9, 10] and type 2 diabetes mellitus [11–13]. For example, low-dose IL-2 treatment in children with recently diagnosed type 1 diabetes resulted in maintained C-peptide production over 1 year [9]. Interestingly, the combination therapy of liraglutide (a glucagon-like peptide 1 [GLP-1] analogue) and anti-IL-21 antibody resulted in enhanced C-peptide secretion over 54 weeks in adults with recent-onset type 1 diabetes [10]. In the field of type 2 diabetes, blockade of IL-1β with IL-1 receptor antagonists reduced inflammation and improved and/or prevented diabetes in a rat model of type 2 diabetes [11], high-fat diet-fed mice [12] and in individuals with type 2 diabetes [13]. Therefore, the identification of novel targets involved in inflammation-related beta cell damage is relevant for the design of novel therapeutic treatments that could prevent or attenuate beta cell failure in both main types of diabetes.

Apolipoprotein L (*APOL*) genes belong to a conserved gene family involved in innate immunity. In humans, this gene family codes for six structurally similar proteins (APOL1–6). *APOL* gene expression is induced by innate immunity-related pathways such as interferons [14–16], polyinosinic:polycytidylic acid [poly(I:C)] in endothelial cells and lipopolysaccharides in podocytes [17], among other cell types. The most well-studied APOL protein is APOL1, and it contains a pore-forming domain (PFD) that consists of a BCL2 homology domain 3 (BH3)-motif, a membrane-address domain (MAD) and a serum resistant-associated (SRA) domain [18, 19]. In addition, APOL1 has a signal peptide domain that enables its secretion extracellularly [20]. Functions associated with the APOL family include lipid transport [21, 22], mitochondria-regulated apoptosis [23, 24] and autophagy [25, 26]. In addition, *APOL1* risk variants cause lysosomal cell death and cell membrane pore formation, as part of their cell defence mechanism against *Trypanosoma* infection [18, 19]. Evidence linking APOLs and diabetes is still very scarce. Croyal et al recently reported a positive association between basal serum APOL1 levels and the risk of developing type 2 diabetes [27]. This was in agreement with previous studies in which an association was found between APOL1 concentrations and plasma triglycerides [28] and/or the metabolic syndrome [29].

While the presence of *APOL* genes has been reported in the pancreas [30], its putative role in beta cell function and the onset or progression of diabetes remains unknown. Here, we have investigated the effect of *APOL* during inflammation-induced beta cell damage.

## Methods

### Human islets and cell lines

Human islets were isolated from pancreases of cadaveric donors through the Eurotransplant multiorgan donation programme. Islets were used for research only if they could not be used for clinical purposes, and if research consent was present, according to Dutch National Laws. Human islets with a purity >80% were cultured in ultra-low attachment plates (Corning ref. 3471, USA) with CMRL 1066 (Corning, 99-663-CV, 5 mmol/l glucose), supplemented with 10% FCS, 2 mmol/l l-glutamine, 100 U/ml penicillin, 100 µg/ml streptomycin, 50 µg/ml gentamycin, 20 µg/ml ciprofloxacin, 10 mmol/l HEPES and 1.2 mg/ml nicotinamide. Donor characteristics are listed in electronic supplementary material (ESM) Table 1. Donors were considered to have type 2 diabetes if a history of diabetes was recorded, and/or the HbA_1c_ level was >48 mmol/mol (6.5%) and there was no indication of other types of diabetes mellitus. Donors were considered to have prediabetes if the HbA_1c_ level was between 39 mmol/mol (5.7%) and 46 mmol/mol (6.4%), according to the ADA guidelines [31].

The EndoC-βH1 cell line [32] was obtained from Univercell Biosolutions (Toulouse, France). Cells were seeded in ECM/fibronectin coated plates and cultured with low glucose DMEM (Invitrogen, USA) supplemented with 10 mmol/l nicotinamide, 5.5 g/ml transferrin, 6.7 ng/ml selenite, penicillin-streptomycin and 50 μmol/l β-mercaptoethanol.

HEK293T cells (ATCC, CRL-3216) were cultured in DMEM + 10% FCS. All cell lines are regularly checked for potential mycoplasma infection in our laboratory.

### Induction of stress conditions

Human islets were exposed for 24 h or 72 h to 1 ng/ml IL-1β (401-ML, R&D systems) + 50 ng/ml IFNγ (285-IF, R&D systems, USA) (cytokines). EndoC-βH1 cells were treated for 24 h or 72 h with 1 ng/ml IL-1β + IFNγ 50 ng/ml (cytokines), 1, 2 or 4 µmol/l baricitinib (a JAK–STAT inhibitor, HY-15315, MedChemExpress, USA) and 1, 2.5 or 5 µmol/l Bay 11-7082 (196871, Merck, Germany) or 50 µg/ml salicylate (S3007, Merck) (NF-κB inhibitors).

### Single-cell RNA-sequencing

We used our single-cell RNA-sequencing Gene Expression Omnibus (GEO; www.ncbi.nlm.nih.gov/geo/) dataset generated from human pancreatic islets treated with beta cell stressors (GSE218316) and performed a differential gene expression analysis of human islet cells treated with IL-1β + IFNγ using a Wilcoxon rank-sum test. We considered genes to be significantly altered if they had an adjusted *p* value (based on Bonferroni correction) <0.05.

### Transcriptomic analysis of type 2 diabetes datasets

Four previously published SMART-seq (GEO GSE83139 [33], GSE81608 [34]) or SMART-seq2 (GEO GSE81547 [35], European Bioinformatics Institute E-MTAB-5061 [36]) scRNA-seq datasets of human pancreatic islets were selected for analysis. Differential gene expression in islets from donors without diabetes and with type 2 diabetes was statistically computed using an unpaired *t* test. See ESM Methods section for details.

### Cell-death staining

Cell death was assessed by using the ReadyProbes Cell Viability Imaging Kit (R37610, Thermo Fisher, USA). EndoC-βH1 cells were incubated with propidium iodide (PI) and Hoechst 33342 for 20 min and imaged with the ImageXpress confocal microscope (Molecular Devices, USA). Blinded quantification was performed with Fiji (2016, https://imagej.net/software/fiji/downloads) by using a nucleus-counting macro.

### RT-qPCR

RNA (250–500 ng) was obtained from human islets and EndoC-βH1 cells by cell lysis with RLT buffer with β-mercaptoethanol according to the manufacturer’s instructions (micro RNeasy kit from Qiagen, Hilden, Germany). Isolated RNA was reverse-transcribed to DNA with M-MLV reverse transcriptase (Invitrogen), oligo(dT)s (Qiagen), dNTP (Promega, USA), DTT (Invitrogen) and RNAse-OUT (ThermoFisher). Quantitative PCR (qPCR) was performed with IQ SYBR green mix (170–8887, Bio-Rad, Hercules, USA). *GAPDH* and *ACTB* were used as housekeeping genes. Amplification and detection were performed by using CFX systems (Bio-Rad) and fold changes were obtained by using the ${2}^{{-\Delta \Delta \mathrm{C}}_{\mathrm{t}}}$ method. Primers are included in ESM Table 2.

### Immunohistochemistry and fluorescence microscopy

Coverslip-cultured EndoC-βH1 cells, isolated human islets or pancreatic tissue samples were fixed in 4% formaldehyde solution and embedded in 2% agar and paraffin. Samples were sectioned into 4 μm slides using the Leica microtome (RM2255, Leica, Germany). Slides were deparaffinised and rehydrated. Blocking was done with 5% normal donkey serum followed by primary and secondary antibody incubations. For nuclei staining, an extra step with 1% Triton X-100 was performed. Immunofluorescence was detected with a Leica microscope (Leica SP8). Antibodies used are listed in ESM Table 3.

### Western blotting

After treatment, approximately 3.000 islet equivalents or 350.000 EndoC-βH1 cells were washed with cold PBS and lysed in RIPA lysis and extraction buffer with 1/100 protease and phosphatase inhibitor (Thermo Fisher). Lysates were passed ten times with a 26-gauge needle and centrifuged to obtain the supernatant. Protein content was measured by BCA protein content kit (Thermo Fisher) and 10–20 μg protein was loaded in 12% mini-PROTEAN TGX gels (Bio-Rad) and transferred to 0.2 µmol/l PVDF membranes (Trans-blot turbo mini 0.2 µmol/l PVDF transfer packs, Bio-Rad). Membranes were blocked with 5% Milk in PBS-Tween for 1 h, incubated with primary antibody overnight at 4°C, and after three washes with PBS-Tween, incubated for 1 h with secondary antibody at room temperature. Primary and secondary antibodies were diluted in the same buffer as the one used for blocking the membrane. Blots were incubated with Supersignal West Pico PLUS Chemiluminescent substrate (Thermo Fisher), visualised with Bio-Rad ChemiDoc Touch (Bio-Rad) and analysed with Image Lab (version 6.1, Bio-Rad). Antibodies used are listed in ESM Table 3.

### Lentivirus-mediated overexpression and knockdown

For overexpression, open reading frames (ORFs) of *APOL1* and *APOL2* in pDONR223 vector were picked from the MISSION TRC3 Human LentiORF Collection (Sigma-Aldrich), amplified by PCR using the following primers: *APOL1* forward (XhoI): TTCTCGAGATGGAGGGAGCTG; *APOL1* reverse (XbaI): AATCTAGAGGCTTGTGTCCACC; *APOL2* forward (XhoI): TTCTCGAGATGAATCCAGAGAGC; *APOL2* reverse (XbaI): AATCTAGATTGGTCTTGGCCTGGC; and subsequently subcloned into pLV-CMV-IRES-puro vector (XhoI/XbaI) for expression. All constructs were verified by sequencing.

The shRNA lentiviral constructs against *APOL1*, *APOL2* and *APOL6* were obtained from the MISSION shRNA library (clones TRCN118633, TRCN83107, TRCN151077, Sigma-Aldrich) and produced as described previously [37].

For transduction, EndoC-βH1 cells were infected with a multiplicity of infection (MOI) of 1 in DMEM/polybrene overnight. After, cells were washed in PBS and replaced with fresh DMEM for 48 h or 72 h, for overexpression or knockdown experiments, respectively.

### siRNA-mediated knockdown

EndoC-βH1 cells were transfected overnight with 30 nmol/l siRNA targeting human *STAT1* (ON-target SMARTpool human si*STAT1*, L-003543-00, Horizon Discovery, Cambridge, UK), or non-target control (ON-TARGETplus Non-targeting siRNAs, D-001810-01, Horizon Discovery). Transfection was performed with Dharmafect transfection reagent (Horizon Discovery) as described in the manufacturer’s instructions. After 48 h, cytokines were added for 24 h.

### Statistics

Results are represented as mean ± SEM. Comparisons were performed by two-tailed student *t* test and a *p* value <0.05 was considered significant.

## Results

### *APOL* genes are expressed in primary human beta cells and upregulated upon inflammation

 We first set out to determine whether *APOL* genes are expressed in primary human pancreatic beta cells using our single-cell RNA-seq dataset from primary human islets exposed to diverse metabolic and inflammatory stressors in parallel: glucose (22 mmol/l) + palmitate (0.5 mmol/l), thapsigargin (0.1 µmol/l), IL-1β (1 ng/ml) + IFNγ (1000 U/ml), IFNα (2000 U/ml), fibroblast growth factor 2 (FGF2; 100 ng/ml) or hypoxia for 24 h and 72 h.

**Fig. 1:**
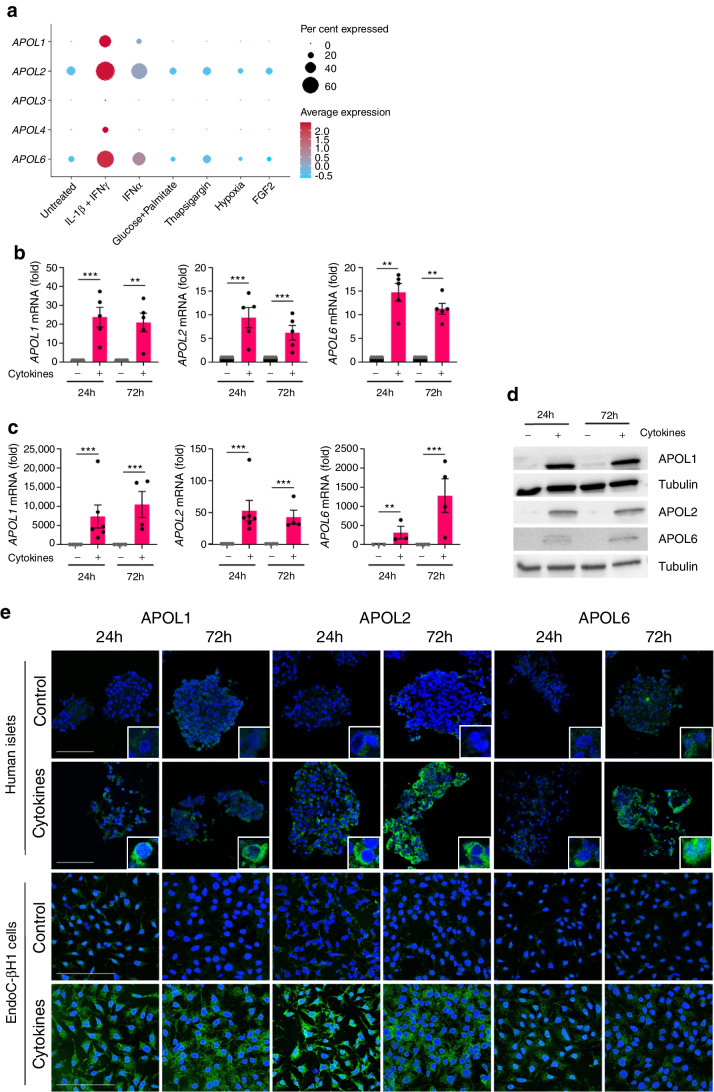
Cytokines increase APOL expression in human islets and beta cells. (**a**) Dotplot from scRNA-seq analysis shows the mean and percentage expression of *APOL* genes in beta cells exposed to different stressors for 24 h and 72 h (merged timepoints). Human islets (**b**, **e**) and EndoC-βH1 cells (**c**, **d**, **e**) were treated with IL-1β + IFNγ (Cytokines) for 24 h and 72 h. (**b**–**e**) Gene and protein expression of APOL1, APOL2 and APOL6 were determined by qPCR (**b**, **c**), western blot (**d**) and immunofluorescence staining (**e**). *ACTB* and *GAPDH* were used to normalise mRNA expression and tubulin was used as loading control for western blots. (**e**) Fluorescence double staining of APOL proteins (green) and DAPI for nuclei (blue) was performed in EndoC-βH1 cells and human islets. Scale bar, 100 µm. (**b**, **c**) Results are the means ± SEM of 3–6 independent experiments; **p<0.01, ***p<0.001, by paired (**b**) or unpaired (**c**) Student’s *t* test

In the untreated condition, *APOL2* and *6* were the most highly expressed in beta cells (Fig. 1a). Upon IL-1β + IFNγ treatment *APOL1*, *APOL2* and *APOL6* gene expression was significantly increased in beta cells, and *APOL2* and *APOL6* was increased in IFNα-treated cells. By contrast, no upregulation of *APOL* genes was found in beta cells exposed to glucose + palmitate, thapsigargin, hypoxia or FGF2 treatments (Fig. 1a and ESM Table 4).

Cytokine-induced expression of *APOL1*, *APOL2* and *APOL6* was validated by qPCR in human islets (Fig. 1b) and EndoC-βH1 cells (Fig. 1c) exposed to IL-1β + IFNγ. In addition, we found a significant upregulation of APOL1, APOL2 and APOL6 protein levels upon inflammation in EndoC-βH1 cells by western blot (Fig. 1d) and by immunostaining in both EndoC-βH1 cells and primary human beta cells (Fig. 1e). Of note, there is no effect of time in culture (with or without cytokines) on *APOL* gene expression (ESM Fig 1a, b and d).

Finally, we assessed the contribution of individual cytokines on the induction of *APOL* genes in beta cells, and we found that *APOL* genes are mainly upregulated by IFNγ (ESM Fig. 1c).

Together, these data reveal that APOL1, APOL2 and APOL6 are specifically upregulated by inflammation in human beta cells.

### Cytokine-induced *APOL* expression is mediated by the JAK–STAT pathway

**Fig. 2:**
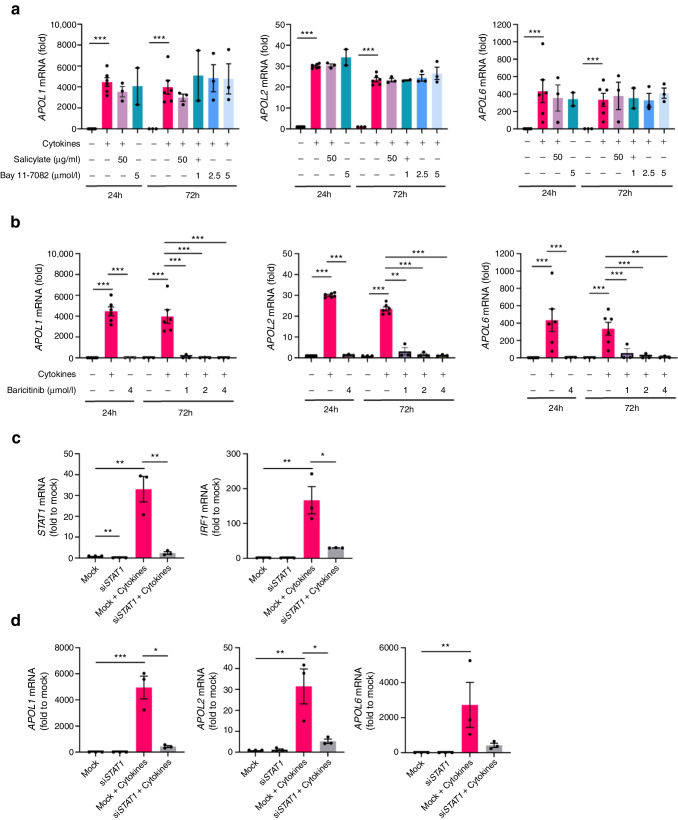
APOL expression is regulated by the JAK–STAT pathway. (**a**, **b**) EndoC-βH1 cells were exposed to IL-1β + IFNγ (Cytokines) for 72 h, alone or in combination with Bay 11-7082/salicylate (NF-κB inhibitors) (**a**) or 4 μmol/l baricitinib (JAK–STAT inhibitor) (**b**). (**c**, **d**) EndoC-βH1 cells were transfected with si*STAT1* or non-targeting siRNA (Mock). After 48 h, the cells were subjected to 24 h cytokine treatment (IL-1β + IFNγ). Gene expression of *STAT1* and *IRF1* (**c**) and *APOL1*, *APOL2* and *APOL6* (**d**) was analysed by qPCR and normalised to housekeeping genes *GAPDH* and *ACTB*. Results are the means ± SEM of 2–5 independent experiments; *p<0.05; **p<0.01, ***p<0.001, by unpaired Student’s *t* test

We next investigated which of the main inflammation-induced signalling pathways mediates the upregulation of *APOL* genes in human beta cells. NF-κB pathway inhibition by salicylate or Bay 11-7082 did not affect cytokine-induced expression of *APOL1*, *APOL2* or *APOL6* (Fig. 2a), but blocked *TNFA* (also known as *TNF*) gene expression as expected (ESM Fig. 2a).

On the other hand, treatment with the JAK–STAT inhibitor baricitinib abolished cytokine-induced *APOL1/APOL2/APOL6* expression significantly both at 24 h and 72 h (Fig. 2b), as well as the IFNγ-response genes *MX1*, *HLA-ABC* and *TNFA* as expected (ESM Fig. 2b).

In order to confirm the involvement of the JAK–STAT pathway, we specifically targeted *STAT1* using siRNA. *STAT1* gene expression was very efficiently downregulated, resulting in reduced expression of its target gene *IRF1* (Fig. 2c). Furthermore, cytokine-induced upregulation of *APOL1* and *2* was strongly prevented upon downregulation of *STAT1* (Fig. 2d).

### *APOL* gene overexpression upregulates ER stress

**Fig. 3:**
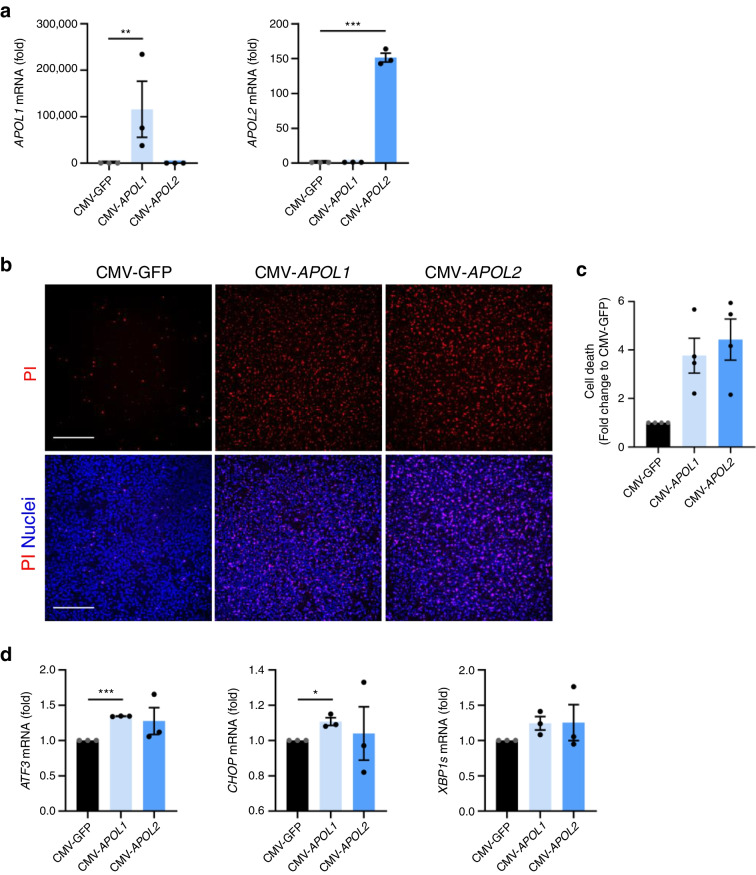
*APOL* overexpression regulates beta cell death. EndoC-βH1 cells were transfected with control empty vector CMV-GFP (black bars) or overexpression constructs for *APOL1* (light blue) or *APOL2* (dark blue). (**a**, **d**) Gene expression was analysed by qPCR and normalised to housekeeping genes *GAPDH* and *ACTB*. (**b**, **c**) Cell death was assessed by propidium iodide (PI; red)/Hoechst (blue) staining (**b**) and quantified with ImageJ (**c**). Scale bar, 500 µm. Results are the means ± SEM of 3–4 independent experiments; *p<0.05, **p<0.01, ***p<0.001 by paired Student’s *t* test compared with the CMV-GFP condition

To investigate the role of *APOL* genes in beta cells, we overexpressed *APOL1* and *APOL2* by lentiviral transduction in EndoC-βH1 cells (Fig. 3a). We observed a trend towards an increased cell death for both *APOL* genes even though the differences failed to reach statistical difference (*p*=0.06 [*APOL1*] and *p*=0.08 [*APOL2*]) (Fig. 3b,c).

In addition, *APOL1* upregulation led to a significant increase of the pro-apoptotic ER stress genes *ATF3* and *CHOP* at 48 h post-transduction (Fig. 3d). Along the same lines, we observed an increase in *ATF3* expression with *APOL1* and *2* overexpression in HEK293T cells (ESM Fig. 3a,b). No change in the protein levels of phosphorylated STAT1 was found upon *APOL1* or *APOL2* overexpression, which indicates overall no change in JAK–STAT pathway activation (ESM Fig. 3c, d). Of note, *ATF3* was also found to be upregulated upon cytokine treatment (ESM Fig. 4a,b,c).

### *APOL1* downregulation prevents cytokine-induced beta cell death and interferon-associated response

**Fig. 4:**
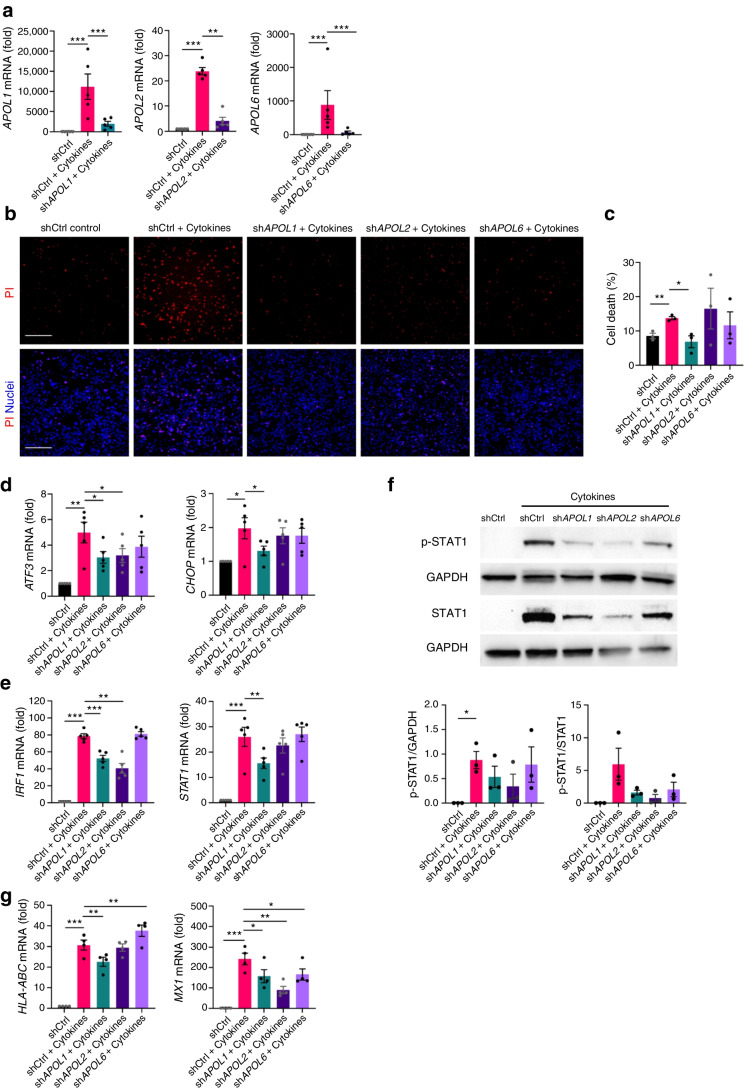
*APOL1* knockdown reduces cytokine-induced cell death and expression of ER stress genes in EndoC-βH1. EndoC-βH1 cells were transfected with shCtrl (black bars) or shRNAs targeting *APOL1* (green), *APOL2* (dark purple) or *APOL6* (light purple) after 72 h of cytokine treatment (IL-1β + IFNγ). Gene expression (**a**, **d**, **e**, **g**) was analysed by qPCR and normalised to housekeeping genes *GAPDH* and *ACTIN* (*n*=4–5). (**b**, **c**) Cell death was assessed by propidium iodide (PI; red)/Hoechst (blue) staining (**b**) and quantified with ImageJ (**c**). Scale bar, 500 µm. (**f**) Protein levels of phospho-STAT1 (Tyr701) (p-STAT1), total STAT1 (STAT1) and GAPDH (as loading control) were analysed by western blotting and quantified. Results are the means ± SEM of 3–5 independent experiments. *p<0.05, **p<0.01, ***p<0.001, by paired Student’s *t* test compared with the indicated condition

Our data so far revealed that *APOL* genes are upregulated upon inflammation and that they could have a detrimental effect on human beta cells. We therefore investigated whether *APOL* gene downregulation could protect against cytokine-induced beta cell damage. We knocked down *APOL1*, *APOL2* or *APOL6* gene expression in cytokine-treated EndoC-βH1 cells leading to a >80% reduced expression of all three genes (Fig. 4a). Strikingly, we found that downregulation of *APOL1* abolished cytokine-induced beta cell death (Fig. 4b,c). This effect was associated with a reduced expression of the cytokine-induced pro-apoptotic ER stress markers *ATF3* and *CHOP* by 39% and 33% via *APOL1* knockdown (Fig. 4d).

Next, we checked the expression of mediators and target genes of the interferon response. We found that cytokine-induced *IRF1* and *STAT1* expression was significantly reduced by *APOL1* knockdown (34% and 50%, respectively) and *IRF1* by *APOL2* knockdown (48%) (Fig. 4e). *APOL6* knockdown did not decrease the expression of the aforementioned genes. In line with gene expression data, exposure to IL-1β + IFNγ increased phosphorylated STAT1, but this was not significantly decreased by *APOL1* and *APOL2* knockdown (Fig. 4f). Finally, *APOL1* downregulation prevented the increase in inflammation-induced expression of target genes *MX1* and *HLA-ABC* by 35% and 27%, respectively (Fig. 4g).

### *APOL* genes are upregulated in human beta cells from donors with type 2 diabetes

**Fig. 5:**
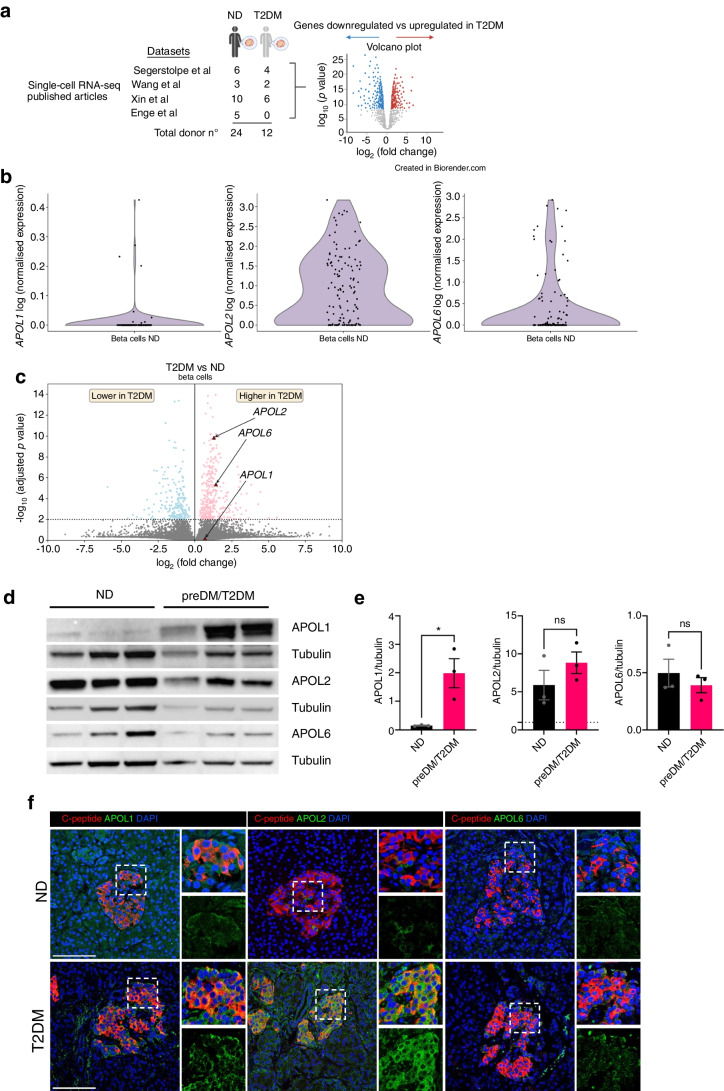
APOL family members are upregulated in islets from donors with type 2 diabetes. (**a**) Schematic representation of the transcriptomic analysis data used to study gene expression in beta cells from donors with type 2 diabetes (T2DM; *n*=12) in comparison with donors without diabetes (no diabetes [ND]; *n*=24); datasets from [33–36]. Created with BioRender.com. (**b**) Violin plots showing gene expression for different *APOL* genes in beta cells from donors without diabetes. (**c**) Volcano plot representing the differential expression of *APOL* genes in donors with type 2 diabetes. (**d**–**f**) APOL1, APOL2 and APOL6 protein expression determination by western blotting (**d**, **e**) in islets from donors without diabetes (ND; *n*=3) and with (pre)diabetes (preDM/T2DM; *n*=3). Fluorescence microscopy (**f**) of APOL1, APOL2 and APOL6 (green), C-peptide (red) and DAPI for nuclei (blue) in islets from donors with type 2 diabetes (T2DM) and without diabetes (ND). Scale bar, 50 µm. Results are the means ± SEM of three independent experiments (**d**, **e**); *p<0.05, by unpaired Student’s *t* test

Finally, we asked whether these findings have any significance in pathophysiological conditions. We merged and analysed four publicly available single-cell transcriptomic datasets of primary human islet cells from donors with type 2 diabetes [33–36] (Fig. 5a, ESM Fig. 5a). *APOL2* and *6* genes were the most highly expressed in beta cells from donors without diabetes, while expression of *APOL1*, *APOL3* and *APOL4* in beta cells was very low and *APOL5* was undetectable (Fig. 5b; ESM Fig. 5b and data not shown).

Furthermore, we found that *APOL 2, 4* and *6* were significantly upregulated in beta cells from donors with type 2 diabetes compared with donors without diabetes (Fig. 5c; ESM Table 4). Interestingly, additional pathway analysis (ESM Fig. 5d,e) showed an enrichment of the interferon pathway in beta cells from the donors with type 2 diabetes analysed compared with donors without diabetes mellitus. Some over-represented genes found were *STAT2*, *IRF9* and *IFI6*, all interferon-regulated genes.

Next, we assessed APOL1, 2 and 6 protein expression by western blot in isolated human islets obtained in our facility (Fig. 5d,e). We observed a significantly higher protein expression of APOL1 by 12.8-fold in islets from three donors with (pre-)diabetes, while APOL2 and APOL6 were not significantly altered in these donors. The increase in APOL1 was confirmed by immunostaining in a pancreas section of another donor with type 2 diabetes (Fig. 5f). APOL2 and APOL6 were also found to be upregulated in beta cells of this donor.

Of note, the upregulation of *APOL* genes was not restricted to beta cells as it was also seen in the single-cell RNA-seq data (see ESM Fig. 5c and ESM Table 5).

Overall, despite inter-donor differences, we found an upregulation of *APOL* genes in beta cells of donors with a history of type 2 diabetes.

## Discussion

Here, we show that *APOL* genes are expressed in human pancreatic beta cells and expression of *APOL1, 2* and *6* is upregulated upon inflammatory conditions. Furthermore, we propose that *APOL* gene upregulation plays a role in the amplification of inflammation in beta cells.

*APOL* gene expression was significantly increased upon cytokine stress, particularly by IFNγ, but not upon other ER stress conditions such as thapsigargin, high glucose or palmitate, which are associated with beta cell dysfunction and/or damage. The results from this study point towards a specific inflammatory regulation of this gene family, in agreement with observations in other cell types where *APOL* genes were also reported as interferon-response genes [15, 17, 26, 37–39].

Moreover, we uncovered that *APOL* genes are upregulated in beta cells from patients with type 2 diabetes mellitus, a condition associated with low-grade inflammation. This is in line with recent studies reporting an association between APOL1 levels in serum and increased risk of type 2 diabetes [27] and the metabolic syndrome [29]. Whether an increase in *APOL* genes can (negatively) influence insulin secretion and contribute to the inflammation-induced beta cell dysfunction that occurs in type 2 diabetes remains to be further investigated. In addition, this study was focused on the role of endogenous *APOL* genes in beta cells. Future studies assessing the impact of exogenous APOL1 on beta cells could contribute to the understanding of the role of elevated APOL1 levels in serum, which are associated with type 2 diabetes risk [27].

We found that *APOL1*, *APOL2* and *APOL6* expression in human beta cells is mediated by the inflammatory JAK–STAT pathway. Other studies have shown that Sp1, IRF1 and IRF2 can bind to the *APOL1* promoter in hepatoma cells [40], and IRF1, IRF2 and STAT2 to the *APOL1* promoter in podocytes and endothelial cells [17]. In addition, the authors of this latter study showed that NF-κB inhibitors downregulate poly(I:C)-induced *APOL1* expression, although NF-κB subunits did not bind to the *APOL1* promoter directly [17]. In our study, we did not observe a role for the NF-κB pathway in regulating *APOL* gene expression. However, we found that downregulation of *APOL1* and *APOL2* in cytokine-treated beta cells was associated with a decrease in IRF1 and STAT1 both at gene and protein levels.

Importantly, ER stress is one of the factors that contributes to the loss of beta cell function in both type 1 diabetes [41] and type 2 diabetes [42], as well as promoting beta cell death [43]. In this study, we showed that *APOL1* or *APOL2* overexpression led to a modest increase in the pro-apoptotic ER stress genes *ATF3* and *CHOP* in EndoC-βH1 cells, and a much stronger induction in HEK293T cells. By contrast, downregulation of *APOL1* gene expression prevented cytokine-induced apoptosis, paralleled by a decrease in ER stress and inflammatory gene expression. A direct contribution of wild type *APOL* genes on induction of ER stress in beta cells will require more evidence, especially in other models, including primary human beta cells. Previous studies have reported that *APOL1* risk variants can induce ER stress in human podocytes [44] and podocyte-like cells from *Drosophila* [45]. Interestingly, inhibition of ER stress likewise prevented APOL1-mediated cell death [44, 45]. *APOL* gene overexpression, especially *APOL1* and *APOL6*, has been reported to promote apoptosis in embryonic kidney cells [46], podocytes [47] and colorectal cancer cell lines [24].

*APOL2* downregulation resulted in a decrease in ER stress and inflammation, while no change was detected in beta cell death. APOL2 has been reported to have an anti-apoptotic role in human bronchial epithelial cells [14] and HeLa cells [15]. Our results also contrast with the work from Liao et al, which showed that *APOL2* knockdown sensitised cells to IFNγ cytotoxicity [14], while Galindo-Moreno et al found no effect of *APOL2* knockdown in IFNγ-induced cell death [15]. Thus the function of *APOL* genes may be partly cell-type specific.

The results from this study indicate that APOL1, APOL2 and APOL6 are overall detrimental. While the role of APOL1 appears to be pro-apoptotic in this and previous work, more studies are needed to decipher the role of other APOLs such as APOL2 and APOL6. Of note, we observed that the *APOL2* expression levels of untreated control beta cells were higher in comparison with other *APOL* genes. Thus, the presence of *APOL2* might be important in maintaining beta cell health up to a certain threshold, after which it could become toxic and eventually trigger cell death. Regarding *APOL6*, earlier studies showed that knockdown inhibited IFNγ-induced apoptosis in atherosclerotic muscle cells [26], implying a negative role for this gene. Yet, we only found a moderate decrease on the levels of ER stress and inflammatory genes upon downregulation of *APOL6* in beta cells exposed to cytokines.

Overall, this study uncovers APOLs as a new gene family that has an implication in beta cell inflammation, and therefore potentially in the onset and/or progression of diabetes mellitus. In conclusion, APOLs appear to be relevant in beta cell health and stress and may be promising targets for reducing progressive beta cell failure.

### Supplementary Information

Below is the link to the electronic supplementary material.

Supplementary file1 (PDF 1.02 MB)

## Data Availability

scRNAseq data generated by our laboratory and used in this study are available in the Gene Expression Omnibus **(**GEO; www.ncbi.nlm.nih.gov/geo/), accession number GSE218316.

## Abbreviations

APOL: Apolipoprotein L
ER stress: Endoplasmic reticulum stress
FGF2: Fibroblast growth factor 2
IRF: Interferon regulatory factor
JAK: Janus kinase
PI: Propidium iodide
qPCR: Quantitative PCR
STAT: Signal transducer and activator of transcription
