# Supplementary material for: Apolipoprotein L genes are novel mediators of inflammation in beta cells
Source: Diabetologia. 2023 Nov 4;67(1):124–36. doi: 10.1007/s00125-023-06033-z (PMC10709252; doi:10.1007/s00125-023-06033-z)
Supplement: Supplementary file 1 — Supplementary file1 (PDF 1.02 MB) [file 125_2023_6033_MOESM1_ESM.pdf]

ELECTRONIC SUPPLEMENTARY MATERIALS

ESM Methods

Transcriptomic analysis of type 2 diabetes datasets

Only adults (21-68 years old) with or without type 2 diabetes were selected. Young children and individuals with type 1 diabetes were excluded from this study. The reads were mapped to the *Homo sapiens* GRCh38 reference genome (Ensembl GTF version 102) using STAR (v2.7.7a) to produce a BAM sequence alignment file. Multimappers were filtered out using Samtools (v1.11). To count the genes, BAM files were split into one BAM file per cell and the genes were counted with FeatureCounts (v2.0.1), where the feature to count was set as the 3'-UTR. Single-cell gene count tables were filtered based on the following criteria: The minimal number of reads per cell was manually selected for each donor and ranged from 3×10^3 to 1×10^5. Similarly, bad quality cells were manually removed by filtering out cells with mitochondrial gene fractions above a certain value (between 0.2 to 0.4). Single-cell RNA-sequencing analysis and visualization were done with the python package Scanpy (v1.7.1). Reads were normalized and log-transformed according to the following formula where n_{gc} is the gene count and N_{tc} the total read count.

$$gc_{normalized} = \ln\left(\frac{n_{gc}}{N_{tc}} \times 10^4 + 1\right)$$

The total counts, the percentage of mitochondrial genes, and the dataset were regressed out to limit their effect during downstream analysis. The data was first dimensionally reduced using principal component analysis (PCA) followed by computing of the Euclidian distance between nearest neighbouring cells on the PCA space using the Batch balanced KNN algorithm. Additional cell filtering was performed by removing unidentifiable cells with the smallest Euclidean distance between other cells above 0.7 and the largest Euclidean distance between cells above 1.5. Cells were projected in a uniform manifold approximation and projection (UMAP) and clustered using the Leiden algorithm [1]. Leiden clusters were annotated by inspecting the expression of known pancreatic marker genes.

References

[1] Traag VA, Waltman L, van Eck NJ (2019) From Louvain to Leiden: guaranteeing well-connected communities. Sci Rep 9(1): 5233. 10.1038/s41598-019-41695-z

ESM Figures and Tables

ESM Table 1

Checklist for reporting human islet preparations used in research (van Tienhoven et al.) Adapted from Hart NJ, Powers AC (2018) Progress, challenges, and suggestions for using human islets to understand islet biology and human diabetes. Diabetologia https://doi.org/10.1007/s00125-018-4772-2.

**Islet preparation**	**1**	**2**	**3**	**4**	**5**	**6**	**7**	**8**
**MANDATORY INFORMATION**								
Unique identifier	Donor 1	Donor 2	Donor 3	Donor 4	Donor 5	Donor 6	Donor 7	Donor 8
Donor age (years)	62	19	69	47	68	51	22	47
Donor sex (M/F)	F	M	M	F	F	M	M	F
Donor BMI (kg/m^2)	29	29	29	28	22	27	25	26
Donor HbA_{1c} or other measure of blood glucose control		38 mmol/mol (5.6%) (last measurement at ICU)	36,2 mmol/mol (5.5%) (last measurement at ICU)	32 mmol/mol (5.1%)			31,8 mmol/mol (5.1%) (last measurement at ICU)	25 mmol/mol (4.4%) (last measurement at ICU)
Origin/source of islets[b]	LUMC	LUMC	LUMC	LUMC	LUMC	LUMC	LUMC	LUMC
Islet isolation centre	LUMC	LUMC	LUMC	LUMC	LUMC	LUMC	LUMC	LUMC
Donor history of diabetes? Please select yes/no from drop down list	No	No	No	No	No	No	No	No
**If Yes, complete the next two lines if this information is available**								

Diabetes duration (years)								
Glucose-lowering therapy at time of death[c]								
**RECOMMENDED INFORMATION**								
Donor cause of death	Circulatory death	Circulatory death	Circulatory death	Circulatory death	Brain death	Circulatory death	Circulatory death	Circulatory death
Warm ischaemia time (h)		0	26 min	17 min	0	10 min	8 min	22 min
Cold ischaemia time (h)								
Estimated purity (%)								
Estimated viability (%)								
Total culture time (h)[d]								
Glucose-stimulated insulin secretion or other functional measurement[e]					.			
Handpicked to purity? Please select yes/no from drop down list								

Additional notes	Used for WB in Fig 5d-e	Used for WB in Fig 5d-e	Used for WB in Fig 5d-e	Used for immuno-fluorescence in Fig 1f 24h panels	Used for immuno-fluorescence in Fig 1f 72h panels	Used for immuno-fluorescence as ND in Fig 5f	Used for qPCR in Fig 1c	Used for qPCR in Fig 1c

**Islet preparation**	**9**	**10**	**11**	**12**	**13**	**14**	**15**	
**MANDATORY INFORMATION**								
Unique identifier	Donor 9	Donor 10	Donor 11	Donor 12	Donor 13	Donor 14	Donor 15	
Donor age (years)	49	53	70	53	69	53	56	
Donor sex (M/F)	M	F	M	F	F	M	M	
Donor BMI (kg/m^2)	26	24	24	28	42	27	46	
Donor HbA_{1c} or other measure of blood glucose control	36 mmol/mol (5.4%) (last measurement at ICU)	31,8 mmol/mol (5.1%)	38 mmol/mol (5.6%)		45 mmol/mol (6.3%)	42.5 mmol/mol (6%) (last measurement at ICU)	42 mmol/mol (6%)	
Origin/source of islets[b]	LUMC	LUMC	LUMC	LUMC	LUMC	LUMC	LUMC	
Islet isolation centre	LUMC	LUMC	LUMC	LUMC	LUMC	LUMC	LUMC	
Donor history of diabetes? Please select yes/no from drop down list	No	No	No	Yes	No	Yes	Yes	
**If Yes, complete the next two lines if this information is available**								

Diabetes duration (years)						1 year		
Glucose-lowering therapy at time of death[c]								

RECOMMENDED INFORMATION								
Donor cause of death	Brain death	Brain death	Circulatory death	Brain death	Circulatory death	Circulatory death	Circulatory death	
Warm ischaemia time (h)	0 min	0 min	21 min	0 min	20 min	19 min	16 min	
Cold ischaemia time (h)							20h	
Estimated purity (%)								
Estimated viability (%)								
Total culture time (h)[d]								
Glucose-stimulated insulin secretion or other functional measurement[e]								
Handpicked to purity? Please select yes/no from drop down list								
Additional notes	Used for qPCR in Fig 1c	Used for qPCR in Fig 1c	Used for qPCR in Fig 1c	Used for WB in Fig 5d-e	Used for WB in Fig 5d-e	Used for WB in Fig 5d-e	Used for immuno-fluorescence as T2DM in Fig 5f	

ESM Table 2: List of human primers

Gene name	5'-> 3'	3'-> 5'
APOL1	AAGAAACAGCGGCTCCACTA	AACGACCATCATCAGGAAGC
APOL2	CAGAGGAGGTTGAGCAGGTC	GTGCCCGCAATTTGTTTACT
APOL6	TTGGTTTGCAAAGGGATGAGGATGA	TCTTTCAATCTGGGAAATTCTCTCA
GAPDH	GGAAGCTTGTCATCAATGG	TGATGATCTTGAGGCTGTTG
ACTB	TGCGTGACATTAAGGAGAAG	TGAAGGTAGTTTCGTGGATG
ATF3	GTGCCGAAACAAGAAGAAGG	TCTGAGCCTTCAGTTCAGCA
CHOP	GACCTGCAAGAGGTCCTGTC	CTCCTCCTCAGTCAGCCAAG
XBP1S	CTGAGTCCGCAGCAGGTG	GAGATGTTCTGGAGGGGTGA
HLA-ABC	AGACAGGACCATCGGAATCT	GTAACCCTTCTTCAGGTGGAAC
MX1	GTGGCATTCAAGGAGTACCTC	GCCTTCGATTCTGGATTCAG
IRF1	TCCGGAGCTGGGCCATTCAC	GCTGAGCTGCCCTTGTTCC
STAT1	CACAAGGTGGCAGGATGTCT	TCCCCGACTGAGCCTGATTA
CXCL8	TCTGGCAACCCTAGTCTGCT	AAACCAAGGCACAGTGGAAC
IL1B	AAGCTGATGGCCCTAAACAG	AGGTGCATCGTGCACATAAG
CXCL10	TCAAGTGGCATAGATGTGGAAGAA	TGGCTCTGCAGGATTTTCATG
TNFA	GTGGGGACTACGACCTGAATG	GGGGCACGATTGTCAAAGATG
APOL1 primers for overexpression	CACTTTTCCTTGGTGTGGGAG	ACCGAGGGGCTTACTTTGAG

ESM Table 3: List of Antibodies

Name	Supplier	Reference	Origin	Dilution
APOL1	Sigma-Aldrich	HPA018885	Rabbit	WB: 1:1000 immunohistochemistry: 1:1000
APOL2	Sigma-Aldrich	HPA001078	Rabbit	WB: 1:2500 immunohistochemistry: 1:250
APOL6	Sigma-Aldrich	HPA029165	Rabbit	WB: 1:500 immunohistochemistry: 1:200
p-STAT1 (Tyr701) (58D6)	Cell signalling	#9167	Rabbit	1:1000
STAT1 (D4Y6Z)	Cell signalling	#14995	Rabbit	1:1000
Tubulin	Cell signalling	#3873	Mouse	1:1.000
GAPDH D16H11 XP	Cell signalling	#5174s	Rabbit	1:1.000
Anti-mouse IgG HRP	Dako Cytomation	P0447	Goat	1:10.000
Anti-rabbit IgG HRP	Dako Cytomation	P0448	Goat	1:10.000

ESM Table 4: Differential expression of APOL genes from scRNA-seq of non-diabetic human islets exposed to stressors

	Gene	avg_log2FC	% Expressed Beta treatment	% Expressed Beta Untreated	p_val_adj
IL1b+IFNg	APOL1	0,910571364	44,8	5,6	2,13E-196
	APOL2	1,21287729	62,4	20,4	7,86E-223
	APOL6	0,746643519	58,1	18,6	1,20E-184
IFNa	APOL2	0,397937852	43,5	20,4	4,70E-62
	APOL6	0,51051017	47,1	18,6	4,50E-92

ESM Table 5: Transcriptomic analysis of type 2 diabetes datasets

	APOL1		*APOL2*		*APOL3*		*APOL4*		*APOL6*	
	**fold change**	**pvals_adj**	**fold change**	**pvals_adj**	**fold change**	**pvals_adj**	**fold change**	**pvals_adj**	**fold change**	**pvals_adj**
**T2D_Ductal cells**	0,96	0,1416	0,79	**0,0068**	1,13	0,7368	1,42	0,6237	0,89	**0,0105**
**T2D_Acinar cells**	0,65	0,5312	2,25	**0,0001**	-18,31	0,5312	24,2	0,5312	1,44	**0,0205**
**T2D_PP-cells**	0,17	1	0,23	0,9393	4,95	0,8209	21,95	0,8143	1,31	0,4841
**T2D_ beta cells**	0,67	0,8485	1,28	**0,0001**	6,73	0,7034	4,29	**0,0314**	1,42	**0,0046**
**T2D_alpha cells**	1,33	0,2673	0,93	**1,14E-11**	3,25	0,467	2,7	0,4515	0,57	**0,0026**
**T2D_Endothelial cells**	-0,64	1	0,85	0,9227	-0,58	1	5,08	0,9227	1,3	0,9227
**T2D_Stellate cells**	-1,04	0,4837	1,34	0,1087	-0,62	0,7882	25,75	0,5919	0,47	0,7716
**T2D_Delta cells**	-0,06	1	0,14	1	1,45	0,96	0,08	1	0,34	1
**T2D_all cells**	0,21	0,5546	1,05	**4,84E-28**	0,18	0,8346	3,63	**0,0001**	0,86	**1,63E-13**

ESM Figure 1

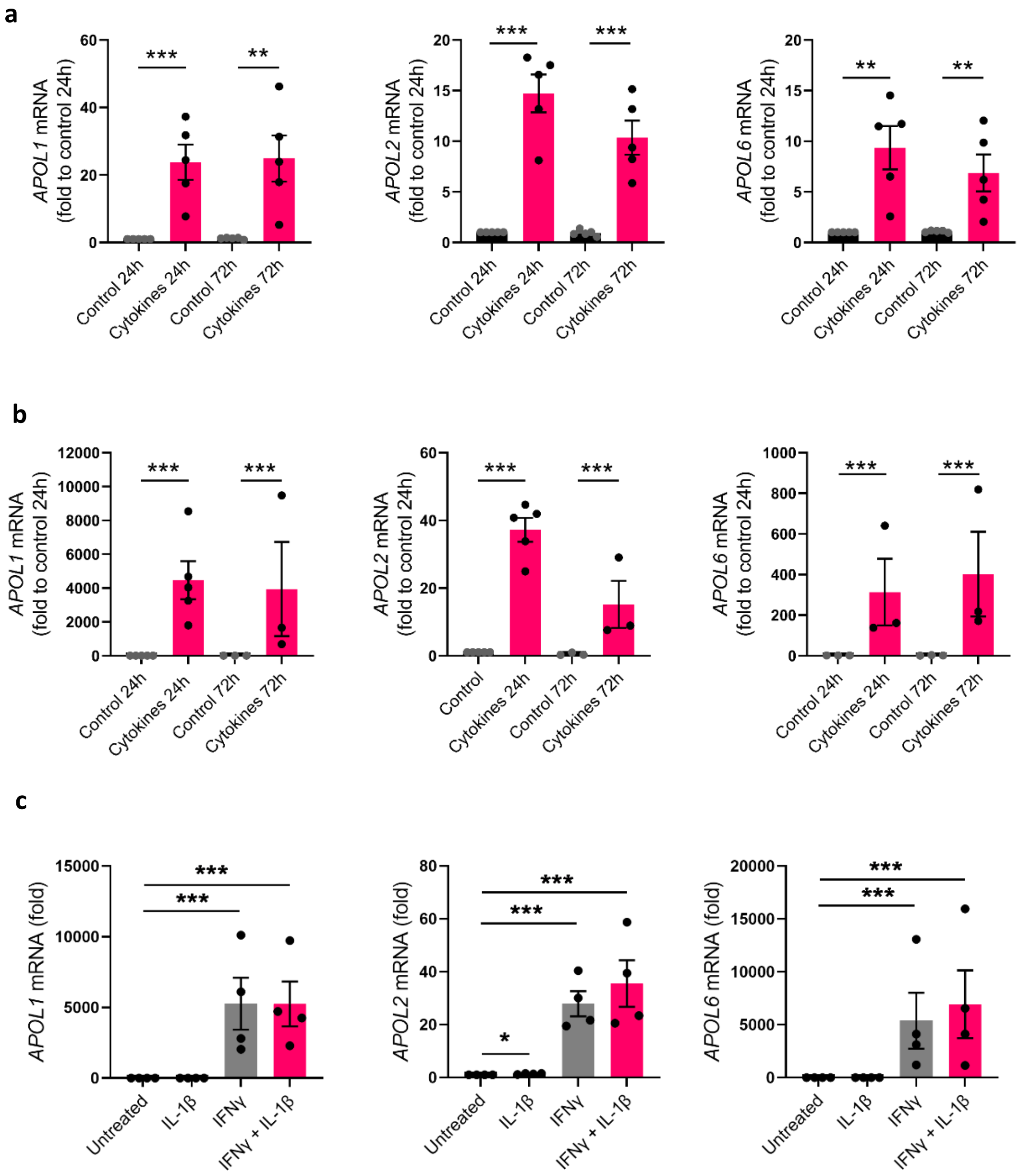

ESM Figure 1: APOL expression is mainly regulated by IFNγ and does not change upon cell culture conditions. Human islets (a) and EndoC-βH1 cells (b,c) were treated with either IL-1β, IFNγ or the combination of IL-1β + IFNγ for 24 h (a-c) or 72 h (a,b). Gene expression of *APOL1*, *APOL2* and *APOL6* was analysed by qPCR and normalized to housekeeping genes *GAPDH* and *ACTB*. Data is normalized to the control 24 h condition (a,b) or untreated control (c) with the 2^{-ddCt} method. Results are the means ± SEM of 3-5 independent experiments; **p <0.01, ***p <0.001, by unpaired Student's t-test

ESM Figure 2

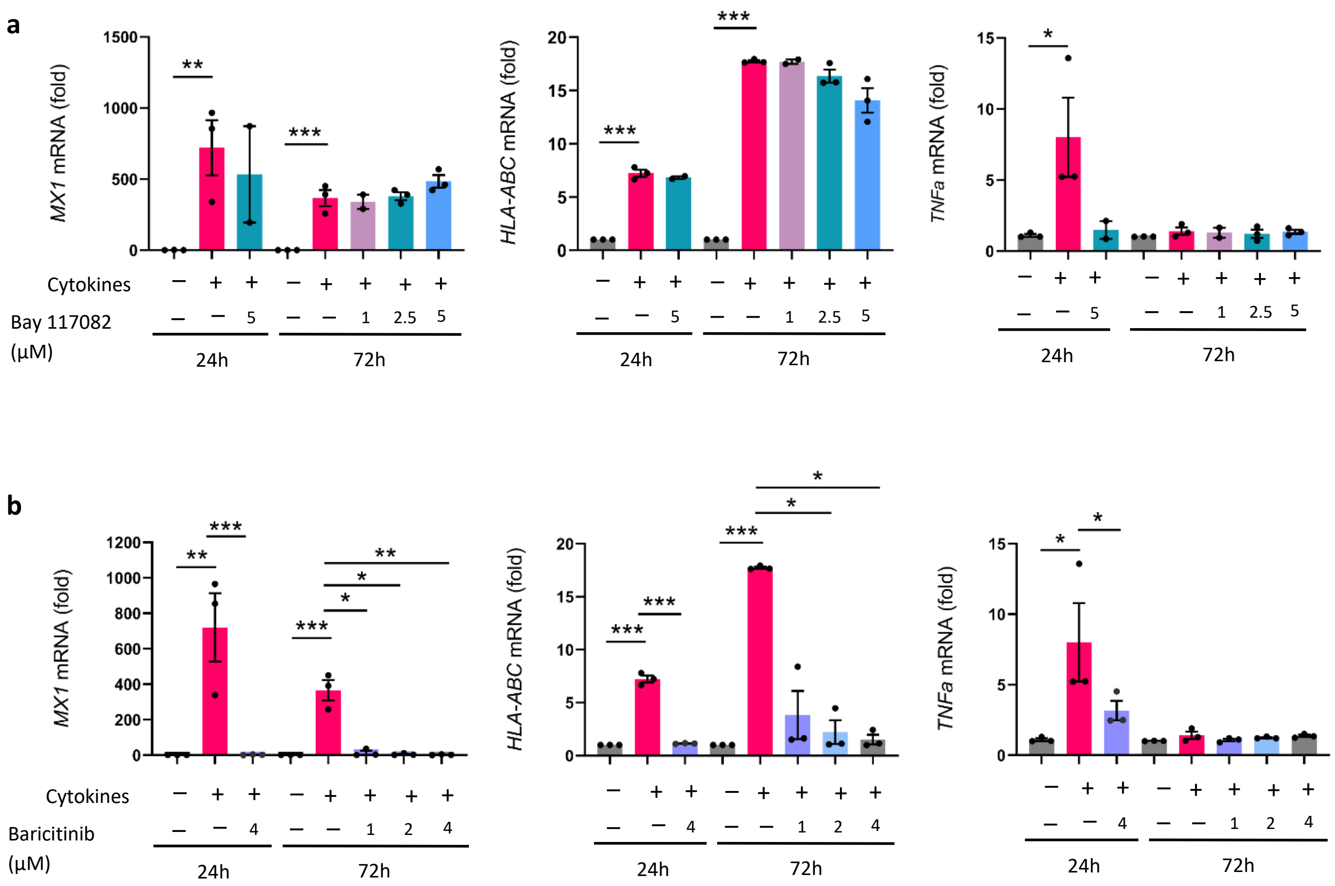

ESM Figure 2: Validation of NFκB and JAK-STAT inhibitors. EndoC-βH1 cells were exposed to IL-1β + IFNγ (Cytokines) for 24 or 72 h, alone or in combination with Bay 11-7082 (NFκB inhibitor) (a) or Baricitinib (JAK-STAT inhibitor) (b). Gene expression of *MX1*, *HLA-ABC* and *TNFA* was analysed by qPCR and normalized to housekeeping genes *GAPDH* and *ACTB*. Results are the means ± SEM of 2-3 independent experiments. *p <0.05, **p <0.01 ***p <0.001, by unpaired Student's t-test

ESM Figure 3

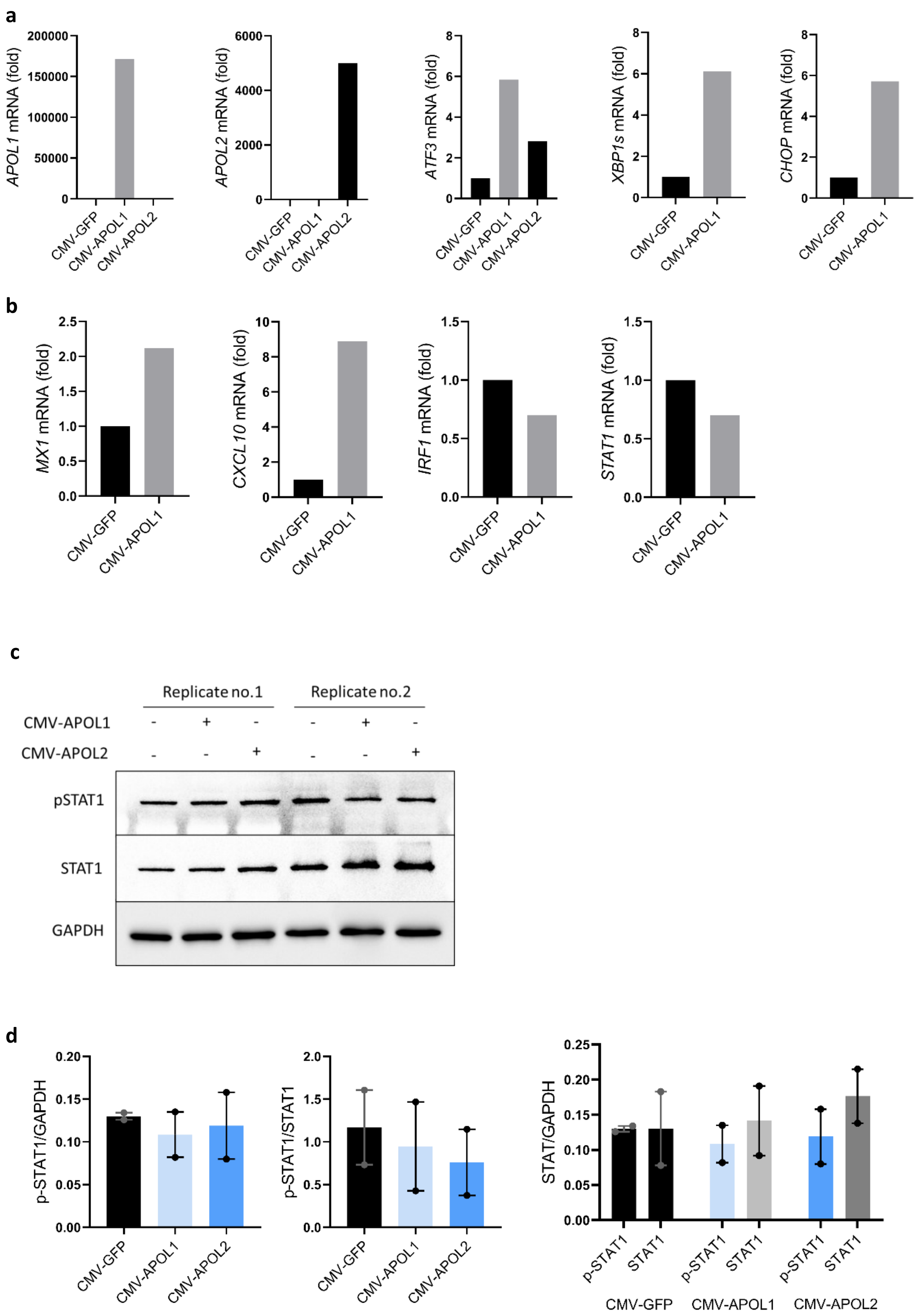

a
APOL1 mRNA (fold)
APOL2 mRNA (fold)
ATF3 mRNA (fold)
XBP1s mRNA (fold)
CHOP mRNA (fold)
CMV-GFP
CMV-APOL1
CMV-APOL2
b
MX1 mRNA (fold)
CXCL10 mRNA (fold)
IRF1 mRNA (fold)
STAT1 mRNA (fold)
c
Replicate no.1
Replicate no.2
CMV-APOL1
CMV-APOL2
pSTAT1
STAT1
GAPDH
d
p-STAT1/GAPDH
p-STAT1/STAT1
STAT/GAPDH
p-STAT1
STAT1

ESM Figure 3: APOL overexpression in HEK293T and EndoC-βH1. HEK293T (a,b) or EndoC-βH1 cells (c,d) were transduced with control empty vector CMV-GFP (black bars) or overexpression constructs targeting *APOL1* or *APOL2*. Gene expression was analysed by qPCR and normalized to housekeeping genes *GAPDH* and *ACTB*. (a) *APOL1*, *APOL2*, and *ATF3* were analysed in all constructs. (b) *XBP1s*, *CHOP*, *MX1*, *CXCL10*, *IRF1* and *STAT1* were analysed for qPCR in CMV-GFP vs. APOL1 OE vector. Results are the means of 1 independent experiment for HEK293T cells. (c) EndoC-βH1 cells were transfected with control empty vector CMV-GFP (black bars) or overexpression constructs targeting *APOL1* (light blue) or *APOL2* (dark blue). Protein levels of phospho-Stat1 (Tyr701) (p-STAT1), total STAT1 (STAT1) and GAPDH (as loading control) were analysed by Western blotting and quantified (d). Results are the means ± SEM of 2 independent experiments.

ESM Figure 4

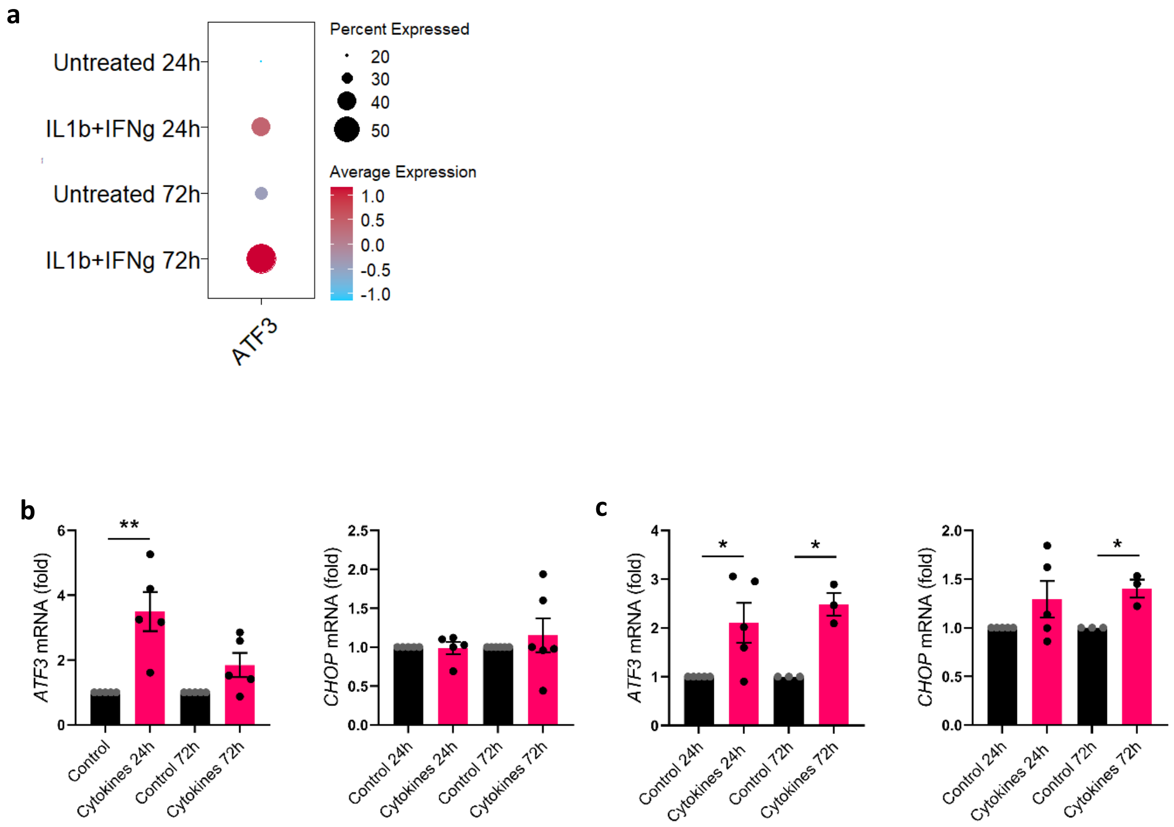

ESM Figure 4: ER-stress is upregulated in beta cells exposed to cytokines. Human islets (a, b) and EndoC-βH1 cells (c) were treated with IL-1β + IFNγ (Cytokines) for 24 h and 72 h. (a) Dotplot from scRNA-seq analysis shows the mean and percentage expression of *ATF3* in beta cells. (b,c) Gene expression of *ATF3* and *CHOP* were determined by qPCR. *ACTB* and *GAPDH* were used to normalize mRNA expression. Results are the means ± SEM of 3-5 independent experiments; *p <0.05, **p <0.01, by paired Student's t-test

ESM Figure 5

a

Dataset	Dataset tag	Dataset accession number	number of donors	number of males	number of females	Healthy	T2D	Sequencing method	year
Segerstolpe et al.	SEG	E-MTAB-5061	10	7	3	6	4	SMART-seq2	2016
Wang et al.	WAN	GSE83139	5	3	2	3	2	SMART-seq	2016
Xin et al.	XIN	GSE81608	16	7	9	10	6	SMART-seq	2016
Enge et al.	ENG	GSE81547	5	3	2	5	0	SMART-seq2	2017
Total			36	20	16	24	12		

b

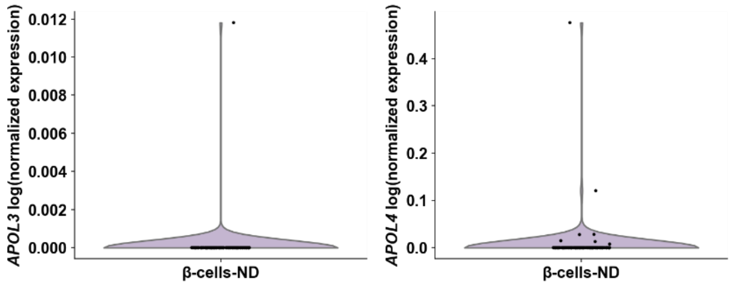

c

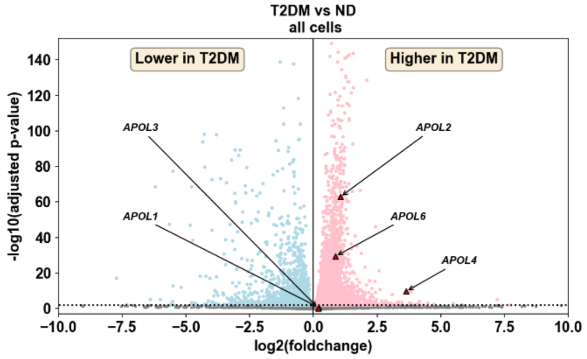

d

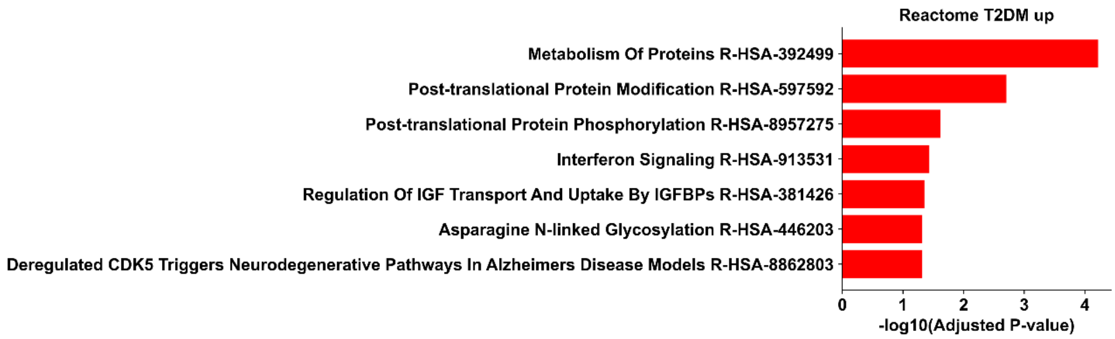

e

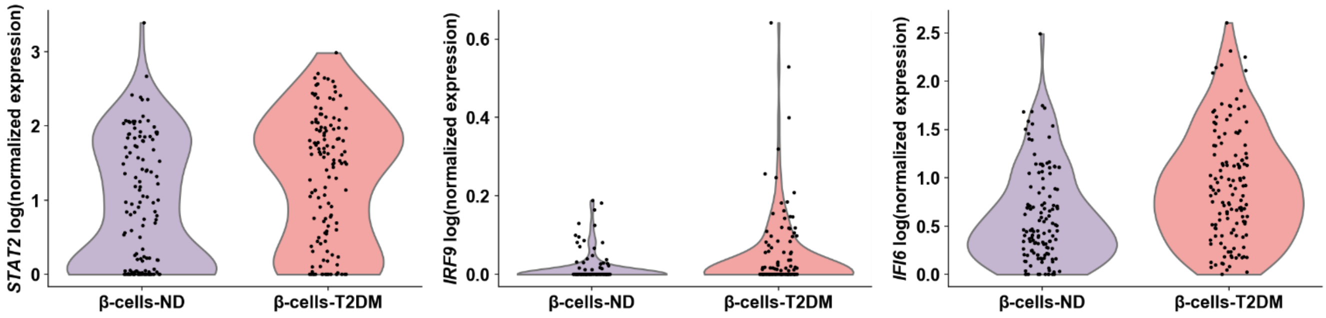

ESM Figure 5: Transcriptomic data on islets from donors with or without type 2 diabetes. (a) Summary information of datasets used. (b) Violin plots showing *APOL3 and APOL4* basal expression in beta cells from donors without diabetes (ND). (c) Volcano plot comparing *APOL* family genes overexpressed in islets from type 2 diabetes mellitus (T2DM) donors in comparison to donors without diabetes. (d,e) Reactome pathway analysis of interferon signalling signature (d) and violin plots (e) for *STAT2*, *IRF9* and *IFI6* expression in beta cells from the same dataset analysis.
